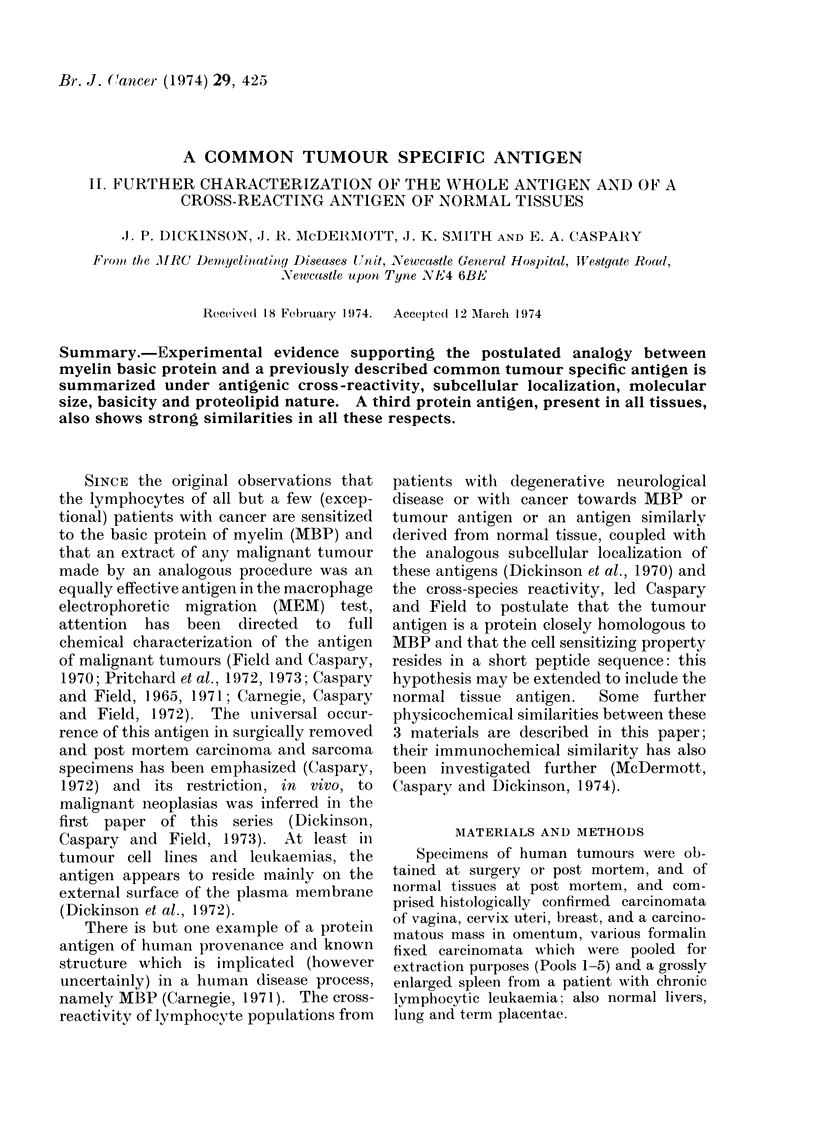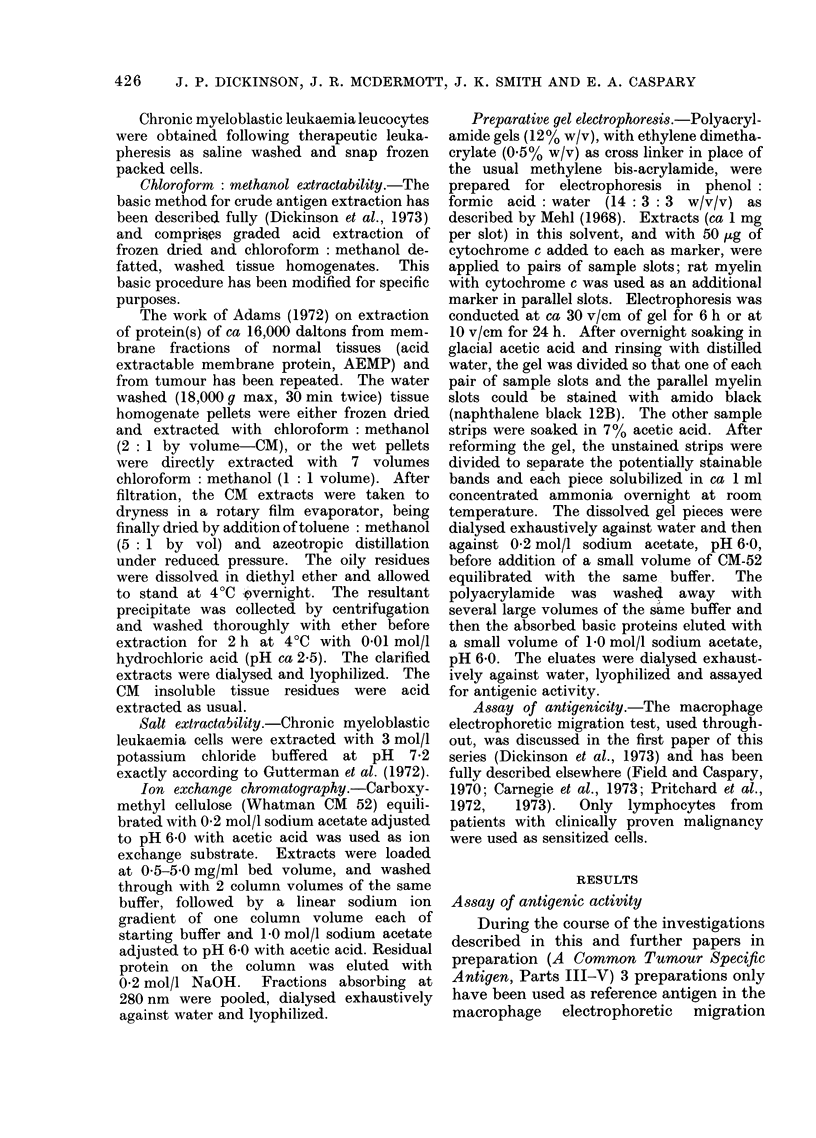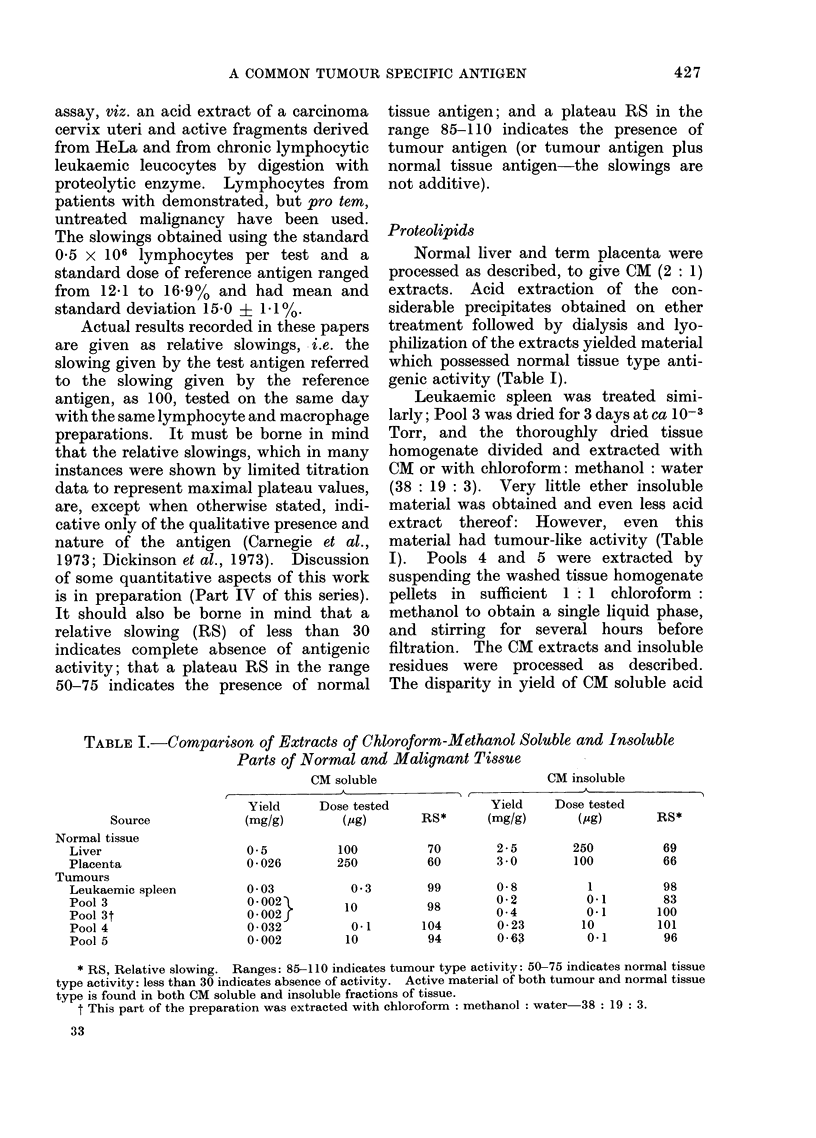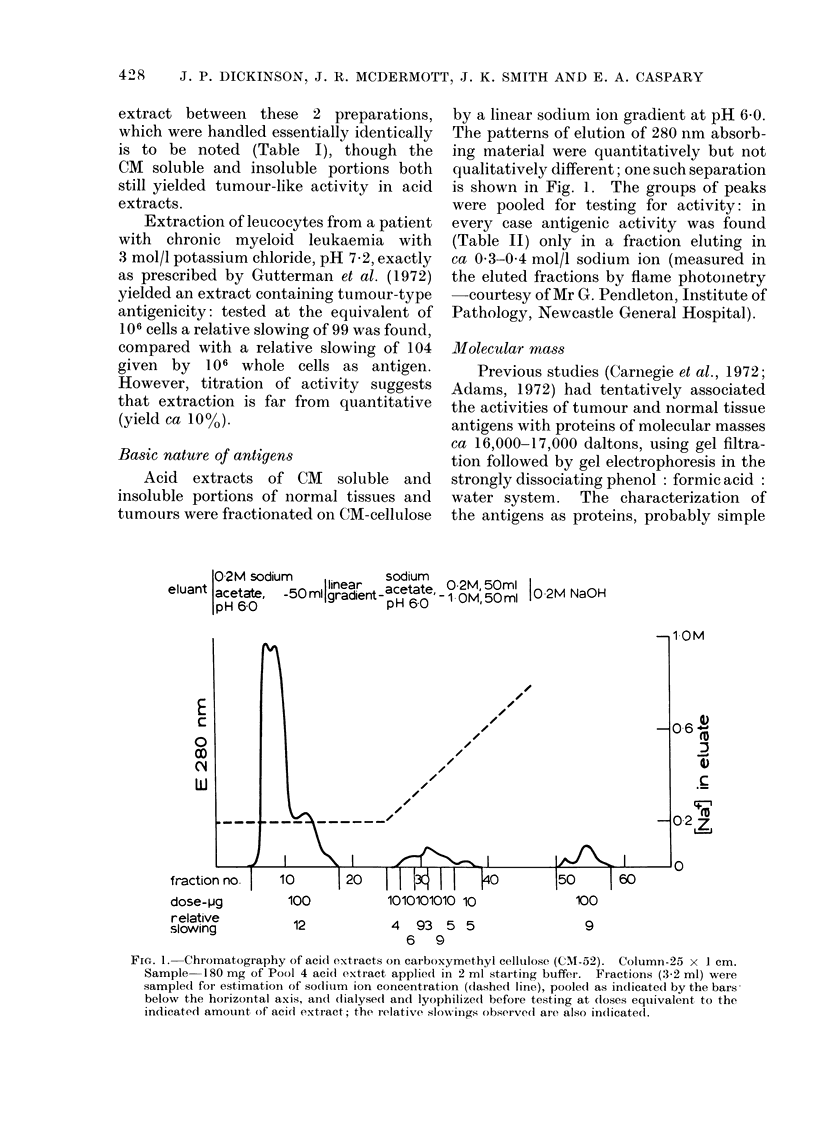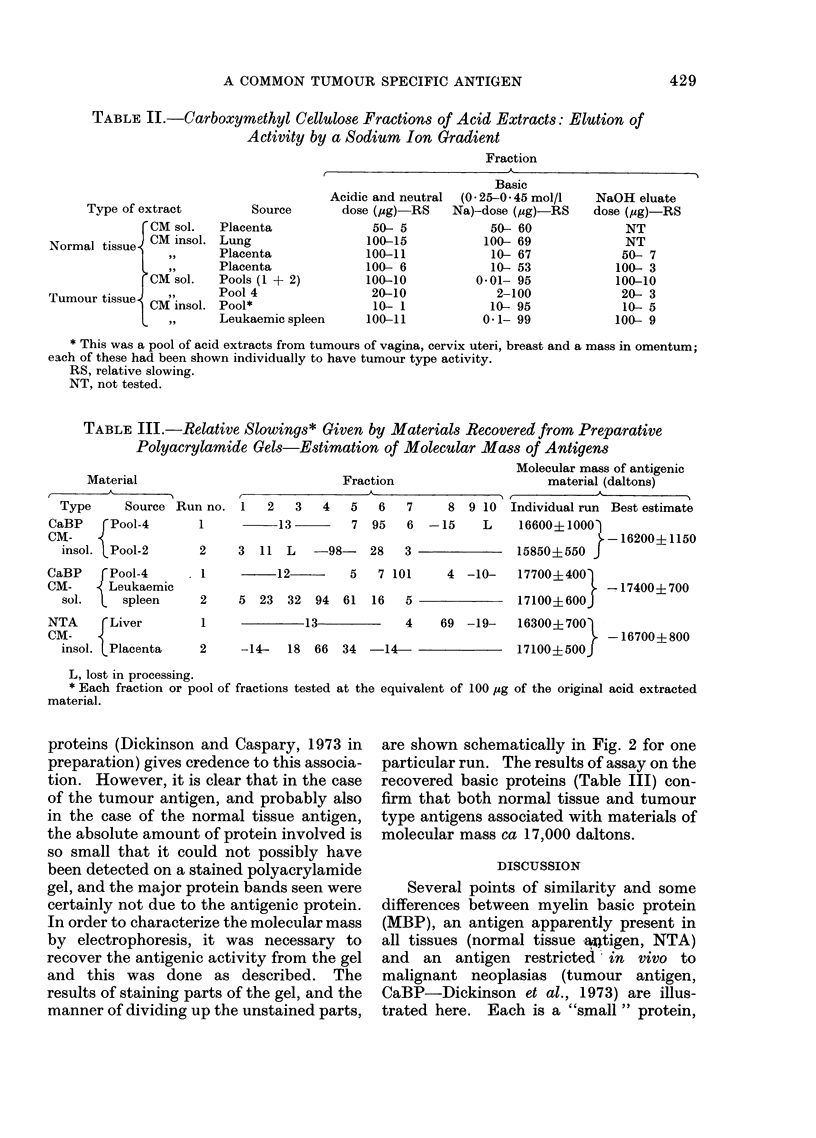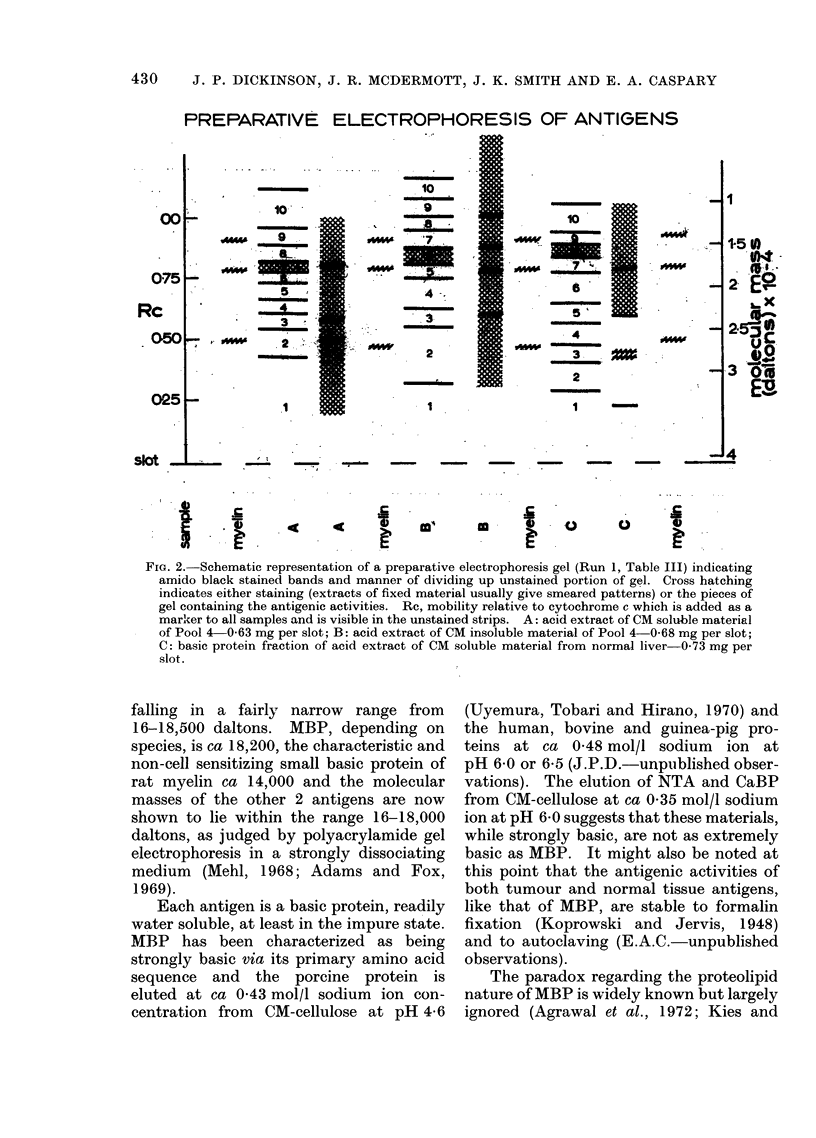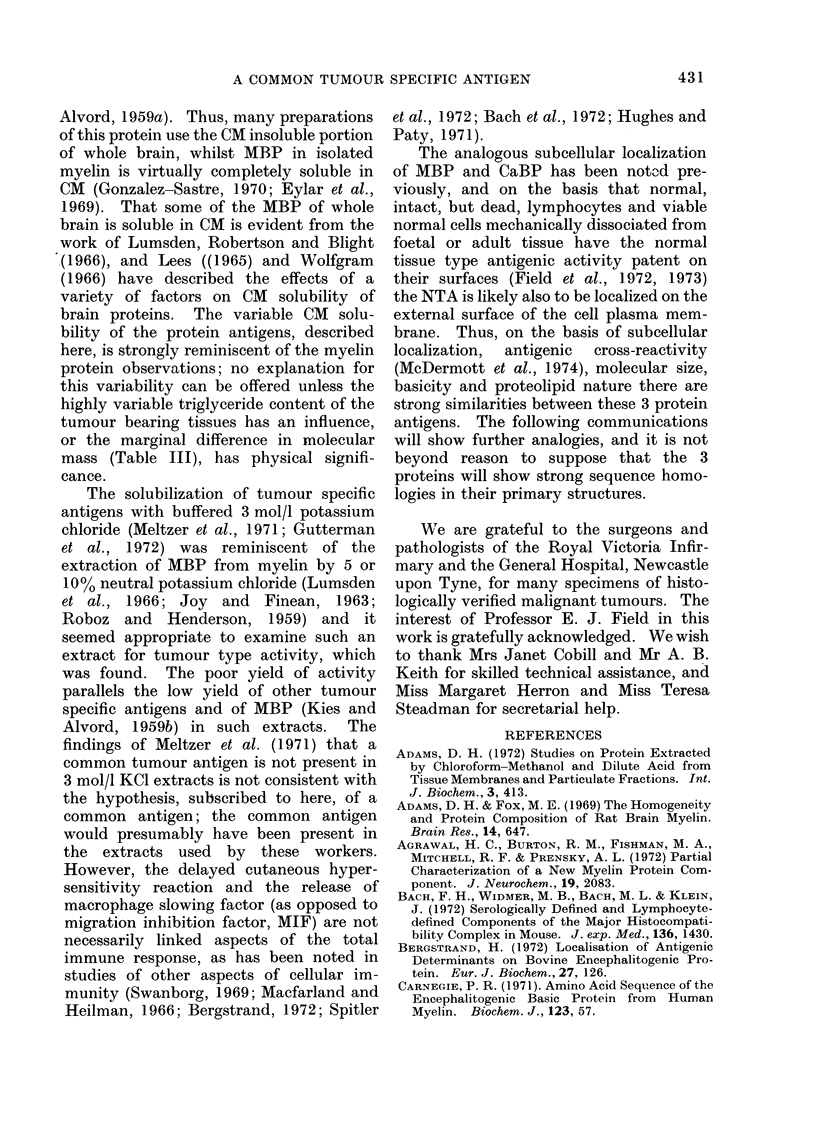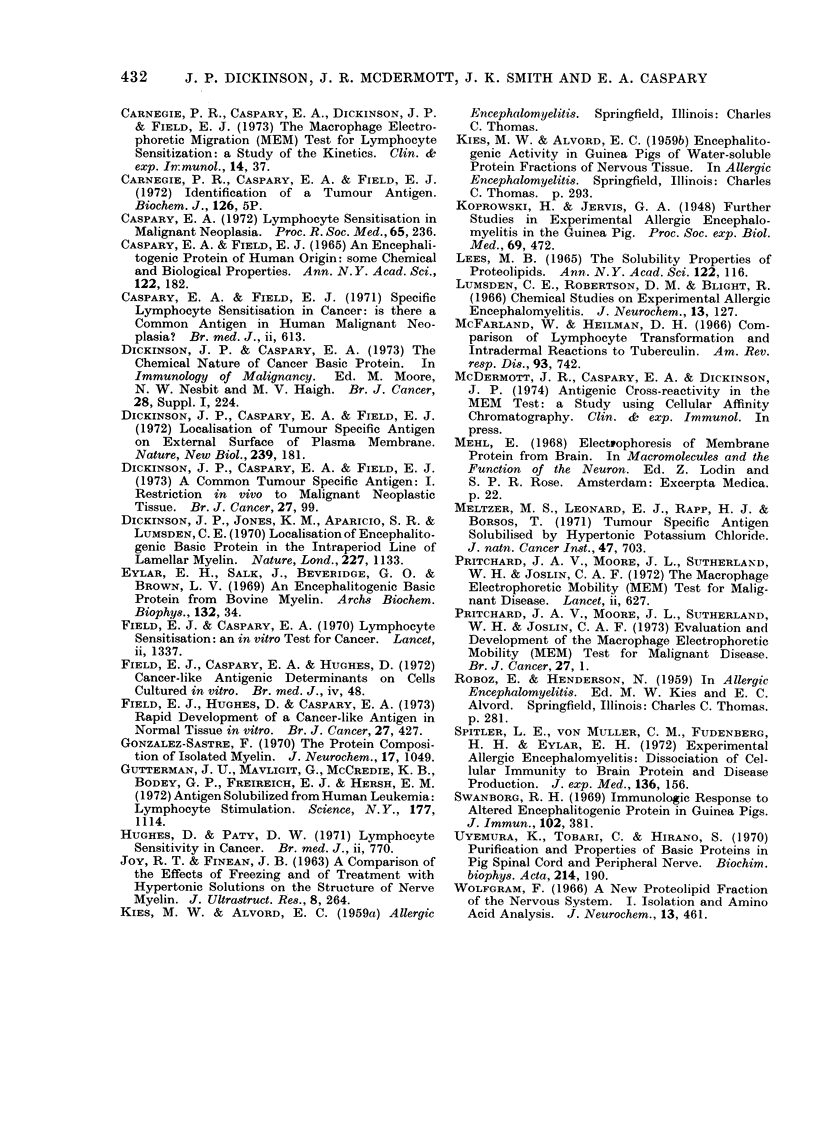# A Common Tumour Specific Antigen

**DOI:** 10.1038/bjc.1974.94

**Published:** 1974-06

**Authors:** J. P. Dickinson, J. R. McDermott, J. K. Smith, E. A. Caspary

## Abstract

Experimental evidence supporting the postulated analogy between myelin basic protein and a previously described common tumour specific antigen is summarized under antigenic cross-reactivity, subcellular localization, molecular size, basicity and proteolipid nature. A third protein antigen, present in all tissues, also shows strong similarities in all these respects.


Br. J. Cancer (1974) 29, 425

### A COMMON TUMOUR SPECIFIC ANTIGEN

### II. FURTHER CHARACTERIZATION OF THE WHOLE ANTIGEN AND OF A CROSS-REACTING ANTIGEN OF NORMAL TISSUES

J. P. DICKINSON, J. R. McDERMOTT, J. K. SMITH AND E. A. CASPARY

*From the MRC Demyelinating Diseases Unit, Newcastle General Hospital, Westgate Road, Newcastle upon Tyne NE4 6BE*

Received 18 February 1974. Accepted 12 March 1974

**Summary.—Experimental evidence supporting the postulated analogy between myelin basic protein and a previously described common tumour specific antigen is summarized under antigenic cross-reactivity, subcellular localization, molecular size, basicity and proteolipid nature. A third protein antigen, present in all tissues, also shows strong similarities in all these respects.**

SINCE the original observations that the lymphocytes of all but a few (exceptional) patients with cancer are sensitized to the basic protein of myelin (MBP) and that an extract of any malignant tumour made by an analogous procedure was an equally effective antigen in the macrophage electrophoretic migration (MEM) test, attention has been directed to full chemical characterization of the antigen of malignant tumours (Field and Caspary, 1970; Pritchard *et al.*, 1972, 1973; Caspary and Field, 1965, 1971; Carnegie, Caspary and Field, 1972). The universal occurrence of this antigen in surgically removed and post mortem carcinoma and sarcoma specimens has been emphasized (Caspary, 1972) and its restriction, *in vivo*, to malignant neoplasias was inferred in the first paper of this series (Dickinson, Caspary and Field, 1973). At least in tumour cell lines and leukaemias, the antigen appears to reside mainly on the external surface of the plasma membrane (Dickinson *et al.*, 1972).

There is but one example of a protein antigen of human provenance and known structure which is implicated (however uncertainly) in a human disease process, namely MBP (Carnegie, 1971). The cross-reactivity of lymphocyte populations from patients with degenerative neurological disease or with cancer towards MBP or tumour antigen or an antigen similarly derived from normal tissue, coupled with the analogous subcellular localization of these antigens (Dickinson *et al.*, 1970) and the cross-species reactivity, led Caspary and Field to postulate that the tumour antigen is a protein closely homologous to MBP and that the cell sensitizing property resides in a short peptide sequence: this hypothesis may be extended to include the normal tissue antigen. Some further physicochemical similarities between these 3 materials are described in this paper; their immunochemical similarity has also been investigated further (McDermott, Caspary and Dickinson, 1974).

### MATERIALS AND METHODS

Specimens of human tumours were obtained at surgery or post mortem, and of normal tissues at post mortem, and comprised histologically confirmed carcinomata of vagina, cervix uteri, breast, and a carcinomatous mass in omentum, various formalin fixed carcinomata which were pooled for extraction purposes (Pools 1–5) and a grossly enlarged spleen from a patient with chronic lymphocytic leukaemia; also normal livers, lung and term placentae.

Chronic myeloblastic leukaemia leucocytes were obtained following therapeutic leukapheresis as saline washed and snap frozen packed cells.

*Chloroform : methanol extractability.*—The basic method for crude antigen extraction has been described fully (Dickinson *et al.*, 1973) and comprises graded acid extraction of frozen dried and chloroform : methanol defatted, washed tissue homogenates. This basic procedure has been modified for specific purposes.

The work of Adams (1972) on extraction of protein(s) of *ca* 16,000 daltons from membrane fractions of normal tissues (acid extractable membrane protein, AEMP) and from tumour has been repeated. The water washed (18,000 *g* max, 30 min twice) tissue homogenate pellets were either frozen dried and extracted with chloroform : methanol (2 : 1 by volume—CM), or the wet pellets were directly extracted with 7 volumes chloroform : methanol (1 : 1 volume). After filtration, the CM extracts were taken to dryness in a rotary film evaporator, being finally dried by addition of toluene : methanol (5 : 1 by vol) and azeotropic distillation under reduced pressure. The oily residues were dissolved in diethyl ether and allowed to stand at 4°C overnight. The resultant precipitate was collected by centrifugation and washed thoroughly with ether before extraction for 2 h at 4°C with 0·01 mol/l hydrochloric acid (pH *ca* 2·5). The clarified extracts were dialysed and lyophilized. The CM insoluble tissue residues were acid extracted as usual.

*Salt extractability.*—Chronic myeloblastic leukaemia cells were extracted with 3 mol/l potassium chloride buffered at pH 7·2 exactly according to Gutterman *et al.* (1972).

*Ion exchange chromatography.*—Carboxymethyl cellulose (Whatman CM 52) equilibrated with 0·2 mol/l sodium acetate adjusted to pH 6·0 with acetic acid was used as ion exchange substrate. Extracts were loaded at 0·5–5·0 mg/ml bed volume, and washed through with 2 column volumes of the same buffer, followed by a linear sodium ion gradient of one column volume each of starting buffer and 1·0 mol/l sodium acetate adjusted to pH 6·0 with acetic acid. Residual protein on the column was eluted with 0·2 mol/l NaOH. Fractions absorbing at 280 nm were pooled, dialysed exhaustively against water and lyophilized.

*Preparative gel electrophoresis.*—Polyacrylamide gels (12% w/v), with ethylene dimethacrylate (0·5% w/v) as cross linker in place of the usual methylene bis-acrylamide, were prepared for electrophoresis in phenol : formic acid : water (14 : 3 : 3 w/v/v) as described by Mehl (1968). Extracts (*ca* 1 mg per slot) in this solvent, and with 50 $\mu$g of cytochrome *c* added to each as marker, were applied to pairs of sample slots; rat myelin with cytochrome *c* was used as an additional marker in parallel slots. Electrophoresis was conducted at *ca* 30 v/cm of gel for 6 h or at 10 v/cm for 24 h. After overnight soaking in glacial acetic acid and rinsing with distilled water, the gel was divided so that one of each pair of sample slots and the parallel myelin slots could be stained with amido black (naphthalene black 12B). The other sample strips were soaked in 7% acetic acid. After reforming the gel, the unstained strips were divided to separate the potentially stainable bands and each piece solubilized in *ca* 1 ml concentrated ammonia overnight at room temperature. The dissolved gel pieces were dialysed exhaustively against water and then against 0·2 mol/l sodium acetate, pH 6·0, before addition of a small volume of CM-52 equilibrated with the same buffer. The polyacrylamide was washed away with several large volumes of the same buffer and then the absorbed basic proteins eluted with a small volume of 1·0 mol/l sodium acetate, pH 6·0. The eluates were dialysed exhaustively against water, lyophilized and assayed for antigenic activity.

*Assay of antigenicity.*—The macrophage electrophoretic migration test, used throughout, was discussed in the first paper of this series (Dickinson *et al.*, 1973) and has been fully described elsewhere (Field and Caspary, 1970; Carnegie *et al.*, 1973; Pritchard *et al.*, 1972, 1973). Only lymphocytes from patients with clinically proven malignancy were used as sensitized cells.

### RESULTS

### *Assay of antigenic activity*

During the course of the investigations described in this and further papers in preparation (*A Common Tumour Specific Antigen*, Parts III–V) 3 preparations only have been used as reference antigen in the macrophage electrophoretic migration assay, *viz.* an acid extract of a carcinoma cervix uteri and active fragments derived from HeLa and from chronic lymphocytic leukaemic leucocytes by digestion with proteolytic enzyme. Lymphocytes from patients with demonstrated, but *pro tem*, untreated malignancy have been used. The slowings obtained using the standard $0.5 \times 10^6$ lymphocytes per test and a standard dose of reference antigen ranged from 12·1 to 16·9% and had mean and standard deviation 15·0 ± 1·1%.

Actual results recorded in these papers are given as relative slowings, *i.e.* the slowing given by the test antigen referred to the slowing given by the reference antigen, as 100, tested on the same day with the same lymphocyte and macrophage preparations. It must be borne in mind that the relative slowings, which in many instances were shown by limited titration data to represent maximal plateau values, are, except when otherwise stated, indicative only of the qualitative presence and nature of the antigen (Carnegie *et al.*, 1973; Dickinson *et al.*, 1973). Discussion of some quantitative aspects of this work is in preparation (Part IV of this series). It should also be borne in mind that a relative slowing (RS) of less than 30 indicates complete absence of antigenic activity; that a plateau RS in the range 50–75 indicates the presence of normal tissue antigen; and a plateau RS in the range 85–110 indicates the presence of tumour antigen (or tumour antigen plus normal tissue antigen—the slowings are not additive).

*Proteolipids*

Normal liver and term placenta were processed as described, to give CM (2 : 1) extracts. Acid extraction of the considerable precipitates obtained on ether treatment followed by dialysis and lyophilization of the extracts yielded material which possessed normal tissue type antigenic activity (Table I).

Leukaemic spleen was treated similarly; Pool 3 was dried for 3 days at *ca* $10^{-3}$ Torr, and the thoroughly dried tissue homogenate divided and extracted with CM or with chloroform: methanol : water (38 : 19 : 3). Very little ether insoluble material was obtained and even less acid extract thereof: However, even this material had tumour-like activity (Table I). Pools 4 and 5 were extracted by suspending the washed tissue homogenate pellets in sufficient 1 : 1 chloroform : methanol to obtain a single liquid phase, and stirring for several hours before filtration. The CM extracts and insoluble residues were processed as described. The disparity in yield of CM soluble acid

TABLE I.—*Comparison of Extracts of Chloroform-Methanol Soluble and Insoluble Parts of Normal and Malignant Tissue*

| | CM soluble | | | CM insoluble | | |
|---|---|---|---|---|---|---|
| Source | Yield (mg/g) | Dose tested (μg) | RS* | Yield (mg/g) | Dose tested (μg) | RS* |
| Normal tissue | | | | | | |
| Liver | 0·5 | 100 | 70 | 2·5 | 250 | 69 |
| Placenta | 0·026 | 250 | 60 | 3·0 | 100 | 66 |
| Tumours | | | | | | |
| Leukaemic spleen | 0·03 | 0·3 | 99 | 0·8 | 1 | 98 |
| Pool 3 | 0·002 } | 10 | 98 | 0·2 | 0·1 | 83 |
| Pool 3† | 0·002 } | | | 0·4 | 0·1 | 100 |
| Pool 4 | 0·032 | 0·1 | 104 | 0·23 | 10 | 101 |
| Pool 5 | 0·002 | 10 | 94 | 0·63 | 0·1 | 96 |

* RS, Relative slowing. Ranges: 85–110 indicates tumour type activity: 50–75 indicates normal tissue type activity: less than 30 indicates absence of activity. Active material of both tumour and normal tissue type is found in both CM soluble and insoluble fractions of tissue.

† This part of the preparation was extracted with chloroform : methanol : water—38 : 19 : 3.

extract between these 2 preparations, which were handled essentially identically is to be noted (Table I), though the CM soluble and insoluble portions both still yielded tumour-like activity in acid extracts.

Extraction of leucocytes from a patient with chronic myeloid leukaemia with 3 mol/l potassium chloride, pH 7·2, exactly as prescribed by Gutterman *et al.* (1972) yielded an extract containing tumour-type antigenicity: tested at the equivalent of $10^6$ cells a relative slowing of 99 was found, compared with a relative slowing of 104 given by $10^6$ whole cells as antigen. However, titration of activity suggests that extraction is far from quantitative (yield *ca* 10%).

*Basic nature of antigens*

Acid extracts of CM soluble and insoluble portions of normal tissues and tumours were fractionated on CM-cellulose by a linear sodium ion gradient at pH 6·0. The patterns of elution of 280 nm absorbing material were quantitatively but not qualitatively different; one such separation is shown in Fig. 1. The groups of peaks were pooled for testing for activity: in every case antigenic activity was found (Table II) only in a fraction eluting in *ca* 0·3–0·4 mol/l sodium ion (measured in the eluted fractions by flame photometry —courtesy of Mr G. Pendleton, Institute of Pathology, Newcastle General Hospital).

*Molecular mass*

Previous studies (Carnegie *et al.*, 1972; Adams, 1972) had tentatively associated the activities of tumour and normal tissue antigens with proteins of molecular masses *ca* 16,000–17,000 daltons, using gel filtration followed by gel electrophoresis in the strongly dissociating phenol : formic acid : water system. The characterization of the antigens as proteins, probably simple

FIG. 1.—Chromatography of acid extracts on carboxymethyl cellulose (CM-52). Column-25 × 1 cm. Sample—180 mg of Pool 4 acid extract applied in 2 ml starting buffer. Fractions (3·2 ml) were sampled for estimation of sodium ion concentration (dashed line), pooled as indicated by the bars below the horizontal axis, and dialysed and lyophilized before testing at doses equivalent to the indicated amount of acid extract; the relative slowings observed are also indicated.

TABLE II.—*Carboxymethyl Cellulose Fractions of Acid Extracts: Elution of Activity by a Sodium Ion Gradient*

| | | | Fraction | | |
|---|---|---|---|---|---|
| Type of extract | | Source | Acidic and neutral dose (μg)—RS | Basic (0·25–0·45 mol/l Na)-dose (μg)—RS | NaOH eluate dose (μg)—RS |
| Normal tissue | CM sol. | Placenta | 50– 5 | 50– 60 | NT |
| | CM insol. | Lung | 100–15 | 100– 69 | NT |
| | ,, | Placenta | 100–11 | 10– 67 | 50– 7 |
| | ,, | Placenta | 100– 6 | 10– 53 | 100– 3 |
| Tumour tissue | CM sol. | Pools (1 + 2) | 100–10 | 0·01– 95 | 100–10 |
| | ,, | Pool 4 | 20–10 | 2–100 | 20– 3 |
| | CM insol. | Pool* | 10– 1 | 10– 95 | 10– 5 |
| | ,, | Leukaemic spleen | 100–11 | 0·1– 99 | 100– 9 |

* This was a pool of acid extracts from tumours of vagina, cervix uteri, breast and a mass in omentum; each of these had been shown individually to have tumour type activity.
RS, relative slowing.
NT, not tested.

TABLE III.—*Relative Slowings* Given by Materials Recovered from Preparative Polyacrylamide Gels—Estimation of Molecular Mass of Antigens*

| Material | | | Fraction | | | | | | | | | | Molecular mass of antigenic material (daltons) | |
|---|---|---|---|---|---|---|---|---|---|---|---|---|---|---|
| Type | Source | Run no. | 1 | 2 | 3 | 4 | 5 | 6 | 7 | 8 | 9 | 10 | Individual run | Best estimate |
| CaBP CM-insol. | Pool-4 | 1 | ——13—— | | | | 7 | 95 | 6 | −15 | | L | 16600±1000 | −16200±1150 |
| | Pool-2 | 2 | 3 | 11 | L | —98— | | 28 | 3 | ———— | | | 15850±550 | |
| CaBP CM-sol. | Pool-4 | 1 | ——12—— | | | | 5 | 7 | 101 | 4 | −10− | | 17700±400 | −17400±700 |
| | Leukaemic spleen | 2 | 5 | 23 | 32 | 94 | 61 | 16 | 5 | ———— | | | 17100±600 | |
| NTA CM-insol. | Liver | 1 | ————13———— | | | | | | 4 | 69 | −19− | | 16300±700 | −16700±800 |
| | Placenta | 2 | −14− | | 18 | 66 | 34 | —14— | | ———— | | | 17100±500 | |

L, lost in processing.
* Each fraction or pool of fractions tested at the equivalent of 100 μg of the original acid extracted material.

proteins (Dickinson and Caspary, 1973 in preparation) gives credence to this association. However, it is clear that in the case of the tumour antigen, and probably also in the case of the normal tissue antigen, the absolute amount of protein involved is so small that it could not possibly have been detected on a stained polyacrylamide gel, and the major protein bands seen were certainly not due to the antigenic protein. In order to characterize the molecular mass by electrophoresis, it was necessary to recover the antigenic activity from the gel and this was done as described. The results of staining parts of the gel, and the manner of dividing up the unstained parts, are shown schematically in Fig. 2 for one particular run. The results of assay on the recovered basic proteins (Table III) confirm that both normal tissue and tumour type antigens associated with materials of molecular mass *ca* 17,000 daltons.

### DISCUSSION

Several points of similarity and some differences between myelin basic protein (MBP), an antigen apparently present in all tissues (normal tissue antigen, NTA) and an antigen restricted *in vivo* to malignant neoplasias (tumour antigen, CaBP—Dickinson *et al.*, 1973) are illustrated here. Each is a "small" protein,

FIG. 2.—Schematic representation of a preparative electrophoresis gel (Run 1, Table III) indicating amido black stained bands and manner of dividing up unstained portion of gel. Cross hatching indicates either staining (extracts of fixed material usually give smeared patterns) or the pieces of gel containing the antigenic activities. Rc, mobility relative to cytochrome *c* which is added as a marker to all samples and is visible in the unstained strips. A: acid extract of CM soluble material of Pool 4—0·63 mg per slot; B: acid extract of CM insoluble material of Pool 4—0·68 mg per slot; C: basic protein fraction of acid extract of CM soluble material from normal liver—0·73 mg per slot.

falling in a fairly narrow range from 16–18,500 daltons. MBP, depending on species, is *ca* 18,200, the characteristic and non-cell sensitizing small basic protein of rat myelin *ca* 14,000 and the molecular masses of the other 2 antigens are now shown to lie within the range 16–18,000 daltons, as judged by polyacrylamide gel electrophoresis in a strongly dissociating medium (Mehl, 1968; Adams and Fox, 1969).

Each antigen is a basic protein, readily water soluble, at least in the impure state. MBP has been characterized as being strongly basic *via* its primary amino acid sequence and the porcine protein is eluted at *ca* 0·43 mol/l sodium ion concentration from CM-cellulose at pH 4·6 (Uyemura, Tobari and Hirano, 1970) and the human, bovine and guinea-pig proteins at *ca* 0·48 mol/l sodium ion at pH 6·0 or 6·5 (J.P.D.—unpublished observations). The elution of NTA and CaBP from CM-cellulose at *ca* 0·35 mol/l sodium ion at pH 6·0 suggests that these materials, while strongly basic, are not as extremely basic as MBP. It might also be noted at this point that the antigenic activities of both tumour and normal tissue antigens, like that of MBP, are stable to formalin fixation (Koprowski and Jervis, 1948) and to autoclaving (E.A.C.—unpublished observations).

The paradox regarding the proteolipid nature of MBP is widely known but largely ignored (Agrawal *et al.*, 1972; Kies and

Alvord, 1959*a*). Thus, many preparations of this protein use the CM insoluble portion of whole brain, whilst MBP in isolated myelin is virtually completely soluble in CM (Gonzalez–Sastre, 1970; Eylar *et al.*, 1969). That some of the MBP of whole brain is soluble in CM is evident from the work of Lumsden, Robertson and Blight (1966), and Lees ((1965) and Wolfgram (1966) have described the effects of a variety of factors on CM solubility of brain proteins. The variable CM solubility of the protein antigens, described here, is strongly reminiscent of the myelin protein observations; no explanation for this variability can be offered unless the highly variable triglyceride content of the tumour bearing tissues has an influence, or the marginal difference in molecular mass (Table III), has physical significance.

The solubilization of tumour specific antigens with buffered 3 mol/l potassium chloride (Meltzer *et al.*, 1971; Gutterman *et al.*, 1972) was reminiscent of the extraction of MBP from myelin by 5 or 10% neutral potassium chloride (Lumsden *et al.*, 1966; Joy and Finean, 1963; Roboz and Henderson, 1959) and it seemed appropriate to examine such an extract for tumour type activity, which was found. The poor yield of activity parallels the low yield of other tumour specific antigens and of MBP (Kies and Alvord, 1959*b*) in such extracts. The findings of Meltzer *et al.* (1971) that a common tumour antigen is not present in 3 mol/l KCl extracts is not consistent with the hypothesis, subscribed to here, of a common antigen; the common antigen would presumably have been present in the extracts used by these workers. However, the delayed cutaneous hypersensitivity reaction and the release of macrophage slowing factor (as opposed to migration inhibition factor, MIF) are not necessarily linked aspects of the total immune response, as has been noted in studies of other aspects of cellular immunity (Swanborg, 1969; Macfarland and Heilman, 1966; Bergstrand, 1972; Spitler *et al.*, 1972; Bach *et al.*, 1972; Hughes and Paty, 1971).

The analogous subcellular localization of MBP and CaBP has been noted previously, and on the basis that normal, intact, but dead, lymphocytes and viable normal cells mechanically dissociated from foetal or adult tissue have the normal tissue type antigenic activity patent on their surfaces (Field *et al.*, 1972, 1973) the NTA is likely also to be localized on the external surface of the cell plasma membrane. Thus, on the basis of subcellular localization, antigenic cross-reactivity (McDermott *et al.*, 1974), molecular size, basicity and proteolipid nature there are strong similarities between these 3 protein antigens. The following communications will show further analogies, and it is not beyond reason to suppose that the 3 proteins will show strong sequence homologies in their primary structures.

We are grateful to the surgeons and pathologists of the Royal Victoria Infirmary and the General Hospital, Newcastle upon Tyne, for many specimens of histologically verified malignant tumours. The interest of Professor E. J. Field in this work is gratefully acknowledged. We wish to thank Mrs Janet Cobill and Mr A. B. Keith for skilled technical assistance, and Miss Margaret Herron and Miss Teresa Steadman for secretarial help.

### REFERENCES

ADAMS, D. H. (1972) Studies on Protein Extracted by Chloroform–Methanol and Dilute Acid from Tissue Membranes and Particulate Fractions. *Int. J. Biochem.*, **3**, 413.

ADAMS, D. H. & FOX, M. E. (1969) The Homogeneity and Protein Composition of Rat Brain Myelin. *Brain Res.*, **14**, 647.

AGRAWAL, H. C., BURTON, R. M., FISHMAN, M. A., MITCHELL, R. F. & PRENSKY, A. L. (1972) Partial Characterization of a New Myelin Protein Component. *J. Neurochem.*, **19**, 2083.

BACH, F. H., WIDMER, M. B., BACH, M. L. & KLEIN, J. (1972) Serologically Defined and Lymphocyte-defined Components of the Major Histocompatibility Complex in Mouse. *J. exp. Med.*, **136**, 1430.

BERGSTRAND, H. (1972) Localisation of Antigenic Determinants on Bovine Encephalitogenic Protein. *Eur. J. Biochem.*, **27**, 126.

CARNEGIE, P. R. (1971). Amino Acid Sequence of the Encephalitogenic Basic Protein from Human Myelin. *Biochem. J.*, **123**, 57.

CARNEGIE, P. R., CASPARY, E. A., DICKINSON, J. P. & FIELD, E. J. (1973) The Macrophage Electrophoretic Migration (MEM) Test for Lymphocyte Sensitization: a Study of the Kinetics. *Clin. & exp. Immunol.*, **14**, 37.

CARNEGIE, P. R., CASPARY, E. A. & FIELD, E. J. (1972) Identification of a Tumour Antigen. *Biochem. J.*, **126**, 5P.

CASPARY, E. A. (1972) Lymphocyte Sensitisation in Malignant Neoplasia. *Proc. R. Soc. Med.*, **65**, 236.

CASPARY, E. A. & FIELD, E. J. (1965) An Encephalitogenic Protein of Human Origin: some Chemical and Biological Properties. *Ann. N.Y. Acad. Sci.*, **122**, 182.

CASPARY, E. A. & FIELD, E. J. (1971) Specific Lymphocyte Sensitisation in Cancer: is there a Common Antigen in Human Malignant Neoplasia? *Br. med. J.*, ii, 613.

DICKINSON, J. P. & CASPARY, E. A. (1973) The Chemical Nature of Cancer Basic Protein. In *Immunology of Malignancy*. Ed. M. Moore, N. W. Nesbit and M. V. Haigh. *Br. J. Cancer*, **28**, Suppl. I, 224.

DICKINSON, J. P., CASPARY, E. A. & FIELD, E. J. (1972) Localisation of Tumour Specific Antigen on External Surface of Plasma Membrane. *Nature, New Biol.*, **239**, 181.

DICKINSON, J. P., CASPARY, E. A. & FIELD, E. J. (1973) A Common Tumour Specific Antigen: I. Restriction *in vivo* to Malignant Neoplastic Tissue. *Br. J. Cancer*, **27**, 99.

DICKINSON, J. P., JONES, K. M., APARICIO, S. R. & LUMSDEN, C. E. (1970) Localisation of Encephalitogenic Basic Protein in the Intraperiod Line of Lamellar Myelin. *Nature, Lond.*, **227**, 1133.

EYLAR, E. H., SALK, J., BEVERIDGE, G. O. & BROWN, L. V. (1969) An Encephalitogenic Basic Protein from Bovine Myelin. *Archs Biochem. Biophys.*, **132**, 34.

FIELD, E. J. & CASPARY, E. A. (1970) Lymphocyte Sensitisation: an *in vitro* Test for Cancer. *Lancet*, ii, 1337.

FIELD, E. J., CASPARY, E. A. & HUGHES, D. (1972) Cancer-like Antigenic Determinants on Cells Cultured *in vitro*. *Br. med. J.*, iv, 48.

FIELD, E. J., HUGHES, D. & CASPARY, E. A. (1973) Rapid Development of a Cancer-like Antigen in Normal Tissue *in vitro*. *Br. J. Cancer*, **27**, 427.

GONZALEZ-SASTRE, F. (1970) The Protein Composition of Isolated Myelin. *J. Neurochem.*, **17**, 1049.

GUTTERMAN, J. U., MAVLIGIT, G., MCCREDIE, K. B., BODEY, G. P., FREIREICH, E. J. & HERSH, E. M. (1972) Antigen Solubilized from Human Leukemia: Lymphocyte Stimulation. *Science, N.Y.*, **177**, 1114.

HUGHES, D. & PATY, D. W. (1971) Lymphocyte Sensitivity in Cancer. *Br. med. J.*, ii, 770.

JOY, R. T. & FINEAN, J. B. (1963) A Comparison of the Effects of Freezing and of Treatment with Hypertonic Solutions on the Structure of Nerve Myelin. *J. Ultrastruct. Res.*, **8**, 264.

KIES, M. W. & ALVORD, E. C. (1959*a*) *Allergic Encephalomyelitis*. Springfield, Illinois: Charles C. Thomas.

KIES, M. W. & ALVORD, E. C. (1959*b*) Encephalitogenic Activity in Guinea Pigs of Water-soluble Protein Fractions of Nervous Tissue. In *Allergic Encephalomyelitis*. Springfield, Illinois: Charles C. Thomas. p. 293.

KOPROWSKI, H. & JERVIS, G. A. (1948) Further Studies in Experimental Allergic Encephalomyelitis in the Guinea Pig. *Proc. Soc. exp. Biol. Med.*, **69**, 472.

LEES, M. B. (1965) The Solubility Properties of Proteolipids. *Ann. N.Y. Acad. Sci.* **122**, 116.

LUMSDEN, C. E., ROBERTSON, D. M. & BLIGHT, R. (1966) Chemical Studies on Experimental Allergic Encephalomyelitis. *J. Neurochem.*, **13**, 127.

MCFARLAND, W. & HEILMAN, D. H. (1966) Comparison of Lymphocyte Transformation and Intradermal Reactions to Tuberculin. *Am. Rev. resp. Dis.*, **93**, 742.

MCDERMOTT, J. R., CASPARY, E. A. & DICKINSON, J. P. (1974) Antigenic Cross-reactivity in the MEM Test: a Study using Cellular Affinity Chromatography. *Clin. & exp. Immunol.* In press.

MEHL, E. (1968) Electrophoresis of Membrane Protein from Brain. In *Macromolecules and the Function of the Neuron*. Ed. Z. Lodin and S. P. R. Rose. Amsterdam: Excerpta Medica. p. 22.

MELTZER, M. S., LEONARD, E. J., RAPP, H. J. & BORSOS, T. (1971) Tumour Specific Antigen Solubilised by Hypertonic Potassium Chloride. *J. natn. Cancer Inst.*, **47**, 703.

PRITCHARD, J. A. V., MOORE, J. L., SUTHERLAND, W. H. & JOSLIN, C. A. F. (1972) The Macrophage Electrophoretic Mobility (MEM) Test for Malignant Disease. *Lancet*, ii, 627.

PRITCHARD, J. A. V., MOORE, J. L., SUTHERLAND, W. H. & JOSLIN, C. A. F. (1973) Evaluation and Development of the Macrophage Electrophoretic Mobility (MEM) Test for Malignant Disease. *Br. J. Cancer*, **27**, 1.

ROBOZ, E. & HENDERSON, N. (1959) In *Allergic Encephalomyelitis*. Ed. M. W. Kies and E. C. Alvord. Springfield, Illinois: Charles C. Thomas. p. 281.

SPITLER, L. E., VON MULLER, C. M., FUDENBERG, H. H. & EYLAR, E. H. (1972) Experimental Allergic Encephalomyelitis: Dissociation of Cellular Immunity to Brain Protein and Disease Production. *J. exp. Med.*, **136**, 156.

SWANBORG, R. H. (1969) Immunologic Response to Altered Encephalitogenic Protein in Guinea Pigs. *J. Immun.*, **102**, 381.

UYEMURA, K., TOBARI, C. & HIRANO, S. (1970) Purification and Properties of Basic Proteins in Pig Spinal Cord and Peripheral Nerve. *Biochim. biophys. Acta*, **214**, 190.

WOLFGRAM, F. (1966) A New Proteolipid Fraction of the Nervous System. I. Isolation and Amino Acid Analysis. *J. Neurochem.*, **13**, 461.